# Decision making under ambiguity and risk in adolescent-onset schizophrenia

**DOI:** 10.1186/s12888-021-03230-1

**Published:** 2021-05-04

**Authors:** Dandan Li, Fengyan Zhang, Lu Wang, Yifan Zhang, Tingting Yang, Kai Wang, Chunyan Zhu

**Affiliations:** 1 Department of Neurology, the First Affiliated Hospital of Anhui Medical University, Hefei, 230022 China; 2 Anhui Province Key Laboratory of Cognition and Neuropsychiatric Disorders, Hefei, 230022 China; 3 Collaborative Innovation Center of Neuropsychiatric Disorders and Mental Health, Hefei, 230022 China; 4 School of Mental Health and Psychological Sciences, Anhui Medical University, Hefei, 230022 China; 5 Children’s Rehabilitation Department, Wuhan Mental Health Center, Wuhan, 430012 China; 6 Institute of Artificial Intelligence, Hefei Comprehensive National Science Center, Hefei, 230022 China

**Keywords:** Adolescent-onset schizophrenia, Decision making, Iowa gambling task, Game of dice task

## Abstract

**Objective:**

Numerous studies have identified impaired decision making (DM) under both ambiguity and risk in adult patients with schizophrenia. However, the assessment of DM in patients with adolescent-onset schizophrenia (AOS) has been challenging as a result of the instability and heterogeneity of manifestations. The Iowa Gambling Task (IGT) and Game of Dice Task (GDT), which are frequently used to evaluate DM respectively under ambiguity and risk, are sensitive to adolescents and neuropsychiatric patients. Our research intended to examine the performance of DM in a relatively large sample of patients with AOS using the above-mentioned two tasks. We also aimed to take a closer look at the relationship between DM and symptom severity of schizophrenia.

**Methods:**

We compared the performance of DM in 71 patients with AOS and 53 well-matched healthy controls using IGT for DM under ambiguity and GDT for DM under risk through net scores, total scores and feedback ration. Neuropsychological tests were conducted in all participants. Clinical symptoms were evaluated by using Positive and Negative Syndrome Scale (PANSS) in 71 patients with AOS. Pearson’s correlation revealed the relationship among total score of DM and clinical and neuropsychological data.

**Results:**

Compared to healthy controls, patients with AOS failed to show learning effect and had a significant difference on the 5th block in IGT and conducted more disadvantageous choices as well as exhibited worse negative feedback rate in GDT. Apart from DM impairment under risk, diminished DM abilities under ambiguity were found related to poor executive function in AOS in the presen**t** study.

**Conclusions:**

Our findings unveiled the abnormal pattern of DM in AOS, mainly reflected under the risky condition, extending the knowledge on the performance of DM under ambiguity and risk in AOS. Inefficient DM under risk may account for the lagging impulse control and the combined effects of developmental disease. In addition, our study demonstrated that the performance on IGT was related to executive function in AOS.

**Supplementary Information:**

The online version contains supplementary material available at 10.1186/s12888-021-03230-1.

## Introduction

Adolescent-onset schizophrenia (AOS) was commonly considered as a chronic  and sever illness with poor prognosis and typical age of onset at 13–18 year [1–4], affecting approximately one child in two hundred [5]. Nearly a third of adult patients with schizophrenia developed their earliest symptoms in the period of children and adolescents,  characterized by high diagnostic stability [5]. According to peviousstudies, the onset of schizophrenia is thought to be basically associated with the combined effects of genetice, developmental, and environmental factors. The adolescent brain was advancing towards maturity, along with the development of the synaptogenesis and myelination [6], suggesting that it may be easier to identify the true etiology of schizophrenia to primarily concern AOS rather than adult schizophrenia [7–9].

In recent year, patients with AOS increasingly suffered with alcoholism, substance abuse and high suicide rates, which may contribute to the premature psychotis symptoms, aggravating manifestations and poor prognosis of AOS [10]. Suicidal behaviors before the first hospitalization occurred in approximate 32% of patients with AOS, and follow-up investigations reported a suicide rate of 12% in the two years after the first admission [11]. Urbanization and social adversity accelerated the occurrence of the harmful behaviors of AOS [12]. Furthermore, risk behaviors were reported to be related with poor DM ability and compelling evidence has proved that DM is one of the strongest predictive factors of risk behaviors [13, 14]. Therefore, it is of great necessity to assess the characteristics of DM in AOS.

DM is the capacity to modulate the advantageous and disadvantageous perception, facilitating rewarded consequences [15, 16], which is generally considered as two types: one is DM under ambiguity (with uncertain probability); the other is DM under risk (with certain probability). DM under ambiguity generally means that decision makers are unaware of the outcomes and probabilities in the beginning, after which they gradually acquire favorable information and optimal choice from the feedback of previous choices [17]. In contrast to DM under ambiguity, DM under risk is associated with explicit information and probabilities which are initailly informed [18]. Moreover, there are representative paradigms for DM under ambiguity and under risk, i.e. IGT (Iowa Gambling Task) [19] and GDT (Game of Dice Task) [20], respectively. IGT, frequently revealing decisions under ambiguity and GDT usually designed to examine DM under a specific risky condition, are both well established and widely applied in the assessment of the neuropsychiatric patients [21–24]. In the past decade, researchers have proceeded with numerous studies to investigate the features of DM deficits in schizophrenia [25–28], which may implicate working memory deficits [29], reward-driven hyperactivities [30, 31], or executive dysfunctions [32]. However, up to date, few consensuses were reached on this issue. Lee et al. (2007) firstly found adult schizophrenia has difficulty in making decisions under ambiguous condition (IGT) but not under risky condition (GDT) [24], whereas, the results of subsequent studies tended to identify the impairments in the schizophrenia under both ambiguous and risky conditions [25, 26, 32, 33]. Consequently, it is of importance to take the above-mentioned DM-related characteristics into account in patients with AOS, in order to reach a general agreement on the DM dysfunctions in the schizophrenia.

With the perspective of DM development, people in adolescence usually preferred to take risks and displayed insensitivity to punishment [34]. Dana et al. (2012) found ambiguous DM abilities progressed in J-shaped curve from 8 to 17 years of age [35], demonstrating children from 8 to10 years of age performed better in IGT than those from 10 to 12 years of age [36], and then the performances on IGT gradually improved in those from 13 to 18 years of age. The compelling evidence indicated accessing the GDT performances may predict the gambling behaviors in adolescents [37]. Studies on DM in healthy adolescents were considered as a foundation, accelerating researches’ investigation on the teenagers with mental or neurological diseases. However, at present, only Kester et al. (2006) investigated the characteristics of DM in patients with AOS under ambiguity using IGT [38]. Patients with AOS exhibited worse during the last two blocks of IGT. They have trouble finding rules to avoid the disadvantageous decks from previous feedback under ambiguity. However, due to a very limited simple size (15 patients with AOS), Kester et al. (2006) may not efficiently demonstrate DM performance in AOS [38]. Meanwhile, to our best knowledge, GDT has not yet been applied to study the characteristics of DM under risk in patients with AOS, though it is widely used in adolescent patients with neurological disease and healthy adolescents [37, 39, 40]. Accordingly, it is of importance and interest to further investigate the performances of DM in AOS in a relatively large sample size, adopting the two different conditions (under ambiguity and under risk).

However, to date, no study has virtually compared the DM performances of AOS and healthy controls under ambiguity and risky respectively at the same time depending on a relatively large sample size, in which the evaluation of the characteristics of DM of AOS would be made more comprehensively and accurately. In the present study, we sought to prove the following hypotheses: (a) patients with AOS demonstrated DM impairments under both ambiguity and risk, utilizing experimental paradigms of IGT and GDT, respectively; Moreover, (b) the performances of DM may be associated with the psychiatric symptoms or cognitive functions in patients with AOS.

## Methods

### Subjects

From November 2013 to January 2019, 71 patients ranged from13 to 18 years old with AOS were recruited from the in-patient and out-patient of the Mental Health Center of Anhui Province in Hefei, China. All the patients met the diagnostic criteria for schizophrenia of the Diagnostic and Statistical Manual of Mental Disorders 4th edition (DSM-IV) [41]. Fifty-three healthy controls were recruited from the local junior-high schools. The two groups were well matched with age, gender, and length of education. Furthermore, those who scored less than or equal to 26 on the Montreal Cognitive Assessment (MoCA) [42], accompanied with anxiety and depression state (Hamilton Anxiety Scale, HAMA> 14, or Hamilton Depression Scale, HAMD> 17) [43, 44], or with a history of substance abuse, neuropsychiatric disorders, or head trauma were excluded from both groups. The Positive and Negative Syndrome Scale (PANSS) [45] was used by trained and experienced psychiatrists to assess the severity of clinical symptoms of patients with AOS. Targets of the assessment are positive subscale score, negative subscale score, general psychopathology subscale score and total scores. Additionally, patients with AOS were at a stable stage of the illness for more than three months. The course of the disease of all patients with AOS is no more than 3 years.

Overall, all the participants in the study completed demographic and psychiatric interviews, underwent the assessment of psychotic symptoms and neuropsychological characteristics. The neuropsychological tests included the MoCA, the Digit Span [46] and the Stroop Color Word [47], assessing the function of the digital and memory and executive function. Detailed demographic, clinical and neuropsychological data are included in Table 1. To avoid the effect of fatigue, all the tests were conducted in two days. All the participants were given the neuropsychological evaluation on the first day, IGT and GDT were performed on the next day in the same order. This study was approved by the Research Ethics Committee of the Anhui Medical University. All participants were right-handed as assessed by Edinburgh Handedness Inventory [48]. All participants and their legal representative gave written informed consent with being given an adequate description and approval of study.

**Table 1:** Demographic and neuropsychological performances in patients with AOS and healthy controls (M ± SD)

Items	Patients with AOS	Healthy controls	***T/x***^***2***^	***P*** value
**Number of participants**	71	53		
**Gender (male/female)**	40/31	23/30	2.034	0.154
**Age (years)**	16.11 ± 1.38	15.80 ± 0.87	1.649	0.102
**Educational year**	9.87 ± 1.47	10.47 ± 0.82	−1.688	0.094
**MoCA**	27.67 ± 1.99	28.6 ± 1.49	−2.495	0.015*
**Stroop Color**	17.02 ± 5.39	12.25 ± 2,78	6700	< 0.001***
**Stroop Word**	20.30 ± 7.02	14.68 ± 3.90	5940	< 0.001***
**Stroop Color&Word**	33.16 ± 13.46	26.34 ± 9.05	3.546	0.001***
**DS-Forward**	7.96 ± 0.25	7.96 ± 0.18	−0.069	0.945
**DS-Backward**	5.45 ± 1.15	6.31 ± 0.84	−4.677	< 0.001***
**PANSS-P**	13.81 ± 6.27			
**PANSS-N**	13.44 ± 6.45			
**PANSS-T**	54.81 ± 14.77			
**CPZeq of total antipsychotic drugs (mg/day)**	441.55 ± 115.76			

Note: *MoCA* Montreal Cognitive Assessment; *Stroop* Stroop Color Word Tasks; *PANSS* Positive and Negative Syndrome Scale. *PANSS - P* PANSS Positive Subscale Score; *PANSS - N* PANSS Negative Subscale Score; *PANSS - T* PANSS Total Score; *DS* The Digital Span Test, *CPZ - eq* Chlorpromazine - equivalent.* *p* < 0.05, *** *p* < 0.001

### Decision-making tasks

#### Iowa gambling task (IGT)

IGT was used to assess DM under ambiguity, which has been modified into the chinesize-computerized version in 2010 [19]. There are 4 decks (A, B, C and D) of cards on the screen, with 100 trials with 2000 € initial capital presented. The participants were asked to select one card among four different decks during each trial without being given any cues, and then they would be informed to win or lose money in each feedback, through which they would find the underlying rule, and then try to maximize monetary outcome. As a specific rule, cards selected from A and B decks were disadvantageous choices under the high-risk condition (an average gain of 100 €), with every 10 selections losing 250 €. The C and D decks were conservative choices, resulting in the gain of win €100 in 10 selections. Eventually, final capital and the total net score were calculated. The net score was the number of decks (C + D)–(A + B). In order to observe the decision strategies of the participants during IGT, 100 trials were divided into five equal blocks for calculating net scores, and subsequently block-wise analysis would be conducted.

#### Game of dice task (GDT)

The computerized GDT was a useful and interesting tool to measure DM under risk. With the participants sitting at the computer, they were given 1000 € as a basic fund and presented with a throwing six-sided dice (numbered: 1–6) [20]. Before the dice was thrown, participants were instructed to try to win as much money as possible during 18 throws. They must choose one from the following four given options:

1. Single number (N1): one-in-six chance to acquire 1000 € gain/loss;
2. Combination of two numbers (N2): two-in-six chance to acquire 500 € gain/loss;
3. Combination of three numbers (N3): three-in-six chance to acquire 200 € gain/loss;
4. Combination of four numbers (N4): four-in-six chance to acquire 100 € gain/loss;

The positive feedback utilization rate: positive feedback numbers (PFN) meant the number of times to choose N3 or N4 option and earn money, and positive feedback switching numbers (PFSN) meant the number of times to earn money after choosing N3 or N4, and then continue choosing N3 or N4 option. The positive feedback utilization rate was calculated by PFSN/PFN, and then turned into percentage (%).

The negative feedback utilization rate: negative feedback numbers (NFN) meant the number of times to choose N1 or N2 option and lose money, and negative feedback switching numbers (NFSN) meant the number of times to lose money after choosing N1 or N2, and then switch to choose N3 or N4 option. The negative feedback utilization rate was calculated by NFSN/NFN, and then turned into percentage (%).

It can be concluded that the higher the return, the higher the risk. N1 and N2 are considered as the high-risky decisions (high-risk options), and N3 and N4 are regarded as the low-risk decisions (low-risk options). After each throw, the feedback of the amount of money will be displayed on the screen. The net score (N3+N4) - (N1-N2) was calculated to investigate GDT performance. The total score was calculated to represent total money earned. Statistical analyses were conducted to calculate the final capital, net score, positive and negative feedback rate, the times to select each one from the four choices and the number of safe/risky decisions.

### Statistical analyses

All statistical analyses were performed by using SPSS, version 23.0 for Windows. All of the variables were examined for normality with the Kolmogorov-smirnov statistic and for homogeneity of variance with the Levene test. Independent sample t-test was conducted to compare the neuropsychological data, background information data, positive and negative feedback rate in GDT, net score and total score in IGT/GDT between patients with AOS and healthy controls. Comparisons with different genders were made by using the Chi-square test. To measure the performance of IGT and GDT, mixed ANOVA was conducted, with the blocks/combination numbers as the within-subject factor, and AOS group and healthy controls group as the between-subjects factors to examine the interaction effect (Blocks/numbers×Group) among these variables. If the interaction effect is significant, analyses of simple effects for group and block interaction were then performed. Post hoc analyses were performed to compare the performances of AOS group and healthy control group in IGT and GDT, using Bonferroni multiple comparison test. Pearson’s correlation analyses were conducted among the total score of DM (IGT and GDT), and clinical and neuropsychological data. For all tests, the threshold of statistical significance was set at *p* < 0.05 (two-tail).

## Results

### Demographic and neuropsychological assessments

The results of demographic materials, clinical evaluation and neuropsychological assessments were shown in Table 1. AOS group and healthy controls group were matched for the age, gender and years of schooling (*p* >  0.05). In neuropsychological tests, AOS group performed significantly worse on the score of the Moca (*p* < 0.05) compared with healthy controls group. With regard to the executive functions on the Stroop tests, AOS group performed significantly worse than healthy controls group (*p* < 0.001). Compared with healthy controls group, AOS group showed lower score on the Digit Span (backward) test (*p* < 0.001).

### Decision-making on IGT

**Fig. 1:**
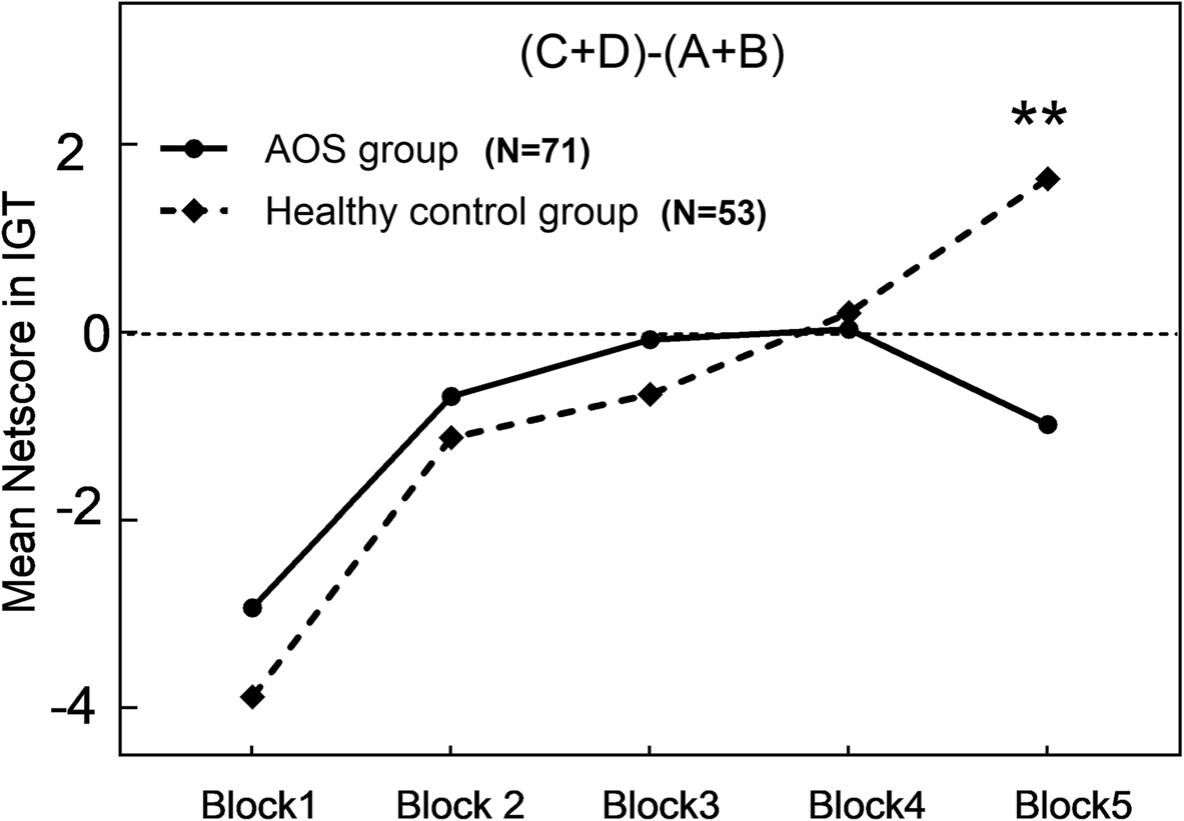
Iowa Gambling Task (IGT) performance across time between patients with AOS and healthy controls. The healthy controls selected more advantageous choices over time, whereas AOS did not differ. Significant net score difference between two groups occurred on the 5th blocks in IGT. Note: ** vs Block 5, *p* < 0.01

The 100 choices were divided into five blocks on average, and the data were analyzed by mixed ANOVA. The block was a within-subjects factor, and the group was a between-subjects factor. For the block, its main effect was significant (*F*_4, 488_ = 16.182, *p* < 0.001, *η*^*2*^ = 0.264), indicating a dynamic change process of IGT. The main effect between groups was not significant (*F*_1, 122_ = 0.150, *p* = 0.699, *η*^*2*^ = 0.001), indicating that there were not differences in the DM strategies between the two groups. There was significant block × group interaction (*F*_4, 488_ = 2.544, *p* = 0.041, *η*^*2*^ = 0.054). The analyses of simple effects for group and block interaction was performed. On the DM performance in each block of IGT, there is no significant difference in the performance between AOS group and healthy control group (*p* >  0.05), except the 5th block (*p* < 0.01). In AOS group, there was no significant difference in the net scores among the five blocks (*p* > 0.05). In normal teenager group, the net score on the 1^st^ block was significantly lower than each net score 2-5^th^ blocks (*p* < 0.001) (Fig. 1). There were no significant differences on total score and net score of IGT between two groups (*p* > 0.05) (Table 2, Fig. 1).

**Table 2:** Decision-making performances of patients with AOS and healthy controls (M ± SD)

Item	Patients with AOS	Healthy Controls concontrolcontrols	***T***	***P*** value	***Conken***^***,***^ ***d***
**IGT**					
**Number of participants**	71	53			
Block1	−2.93 ± 4.80	−3.87 ± 4.30	−1.189	> 0.999	0.176
Block2	−0.67 ± 3.93	−1.12 ± 4.69	− 0.556	> 0.999	0.082
Block3	−0.077 ± 5.24	−0.658 ± 6.126	− 0.730	> 0.999	0.108
Block4	0.038 ± 5.89	0.20 ± 5.91	−0.211	> 0.999	0.031
Block5	−0.98 ± 6.56	1.63 ± 5.64	−3.287	0.005**	0.487
Total net score	−4.63 ± 13.79	−2.53 ± 14.97	−0.988	0.325	0..073 .0883
Total score	1441.18 ± 458.94	1521.34 ± 444.65	−1.194	0.234	0.088
**GDT**					
Number 1(N1)	3.81 ± 3.61	0.98 ± 1.49	5.954	< 0.001***	0.912
Number 2(N2)	3.26 ± 2.3	3.92 ± 3.01	−1.376	0.978	0.211
Number 3(N3)	4.47 ± 2.40	5.90 ± 2.88	−3.002	0.046*	0.459
Number 4(N4)	6.39 ± 3.89	7.18 ± 4.23	−1.082	0.638	0.255
Total net score	3.77 ± 8.07	8.18 ± 7.12	−3.164	0.002**	0.290
Total score	− 2263.38 ± 3392.43	247.16 ± 1925.79	−5.211	< 0.001***	0.469
Use of negative^a^(%) feedback (%)	63.53 ± 27.68	75.19 ± 27.64	−2.321	0.022*	0.209
Use of positive^b^(%) feedback (%)	61.29 ± 32.87	69.64 ± 25.15	−1.593	0.114	0.143

*Note:* effect size: small effect, ≤ 0.30; medium effect, 0.31–0.50; large effect, > 0.50

*p < 0.05, **p < 0.01, *** p < 0.001

### Decision-making on GDT

**Fig. 2:**
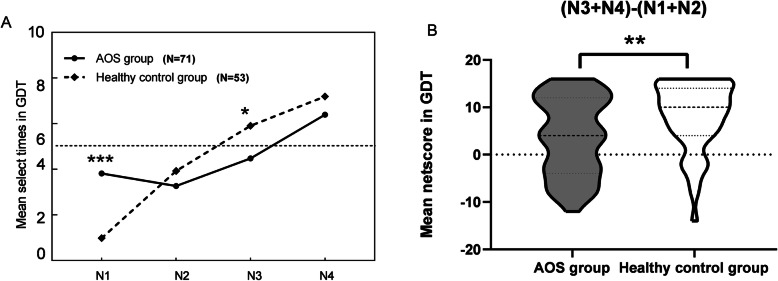
Performances on the Game of Dice in AOS and healthy controls. (A) AOS group was more likely to choose high risk options (N1 and Risk options), whereas the healthy controls preferred to choose the low risk options (N3 and low risk options). Note: *** vs N1, *p* < 0.001, ** vs N3, *p* < 0.01. (B) Group comparisons revealed that means of the net score in AOS were lower than those in the healthy controls Note: **, *p* < 0.01

The data were analyzed by mixed ANOVA. The combination number was the within-subject factor, and the group was a between-subjects factor. For the combination number, its main effect was significant (*F*_3, 366_ = 34.485, *p* < 0.001, *η*^*2*^ = 0.397). The main effect between groups was not significant (*F*_1, 122_ = 1.356, *p* = 0.264, *η*^*2*^ = 0.011). There was significant combination number×group interaction (*F*_3, 366_ = 8.761, *p* < 0.001, *η*^*2*^ = 0.221). The analysis of simple effects for combination number and block interaction was performed. Both AOS group and healthy control group showed higher frequency of choices in the single numbers and three numbers (*p* < 0.01). There was no significant difference between the two groups in two numbers and four numbers in GDT (*p* > 0.05). Significant differences existed between the two groups in the low and high risk options (*p* < 0.01). As shown in Table 2, the total score and net score of patients with AOS in GDT were significantly lower than those of healthy controls (*p* < 0.01). Although the difference in the use of positive feedback did not reach statistical significance (*p* > 0.05), there was a significant difference in the use of negative feedback between the two groups (*p* < 0.05) (Fig. 2).

### Additional analyses on the performance of DM

With including cognitive functions as covariates, no significant effect was found for IGT (*p* > 0.05), while there is a significant interaction effect on GDT (*p* < 0.05). All participates were divided in two groups based on their age: 13–15 years old group and 15–18 years old group. With the gender and education years as covariates, no significant effect was found on either IGT or GDT in 13–15 years old group (*p* > 0.05), while significant interaction effects on both IGT and GDT were observed in 15–18 years old group (*p* < 0.05). Beyond that, we conducted an analysis on effect of gender on either IGT or GDT performances respectively in the AOS. There was no significant interaction effect one IGT, as well as no significant number × gender interaction in the GDT (*p* > 0.05) (see details in the Supplementary Materials).

### Correlational analyses

**Fig. 3:**
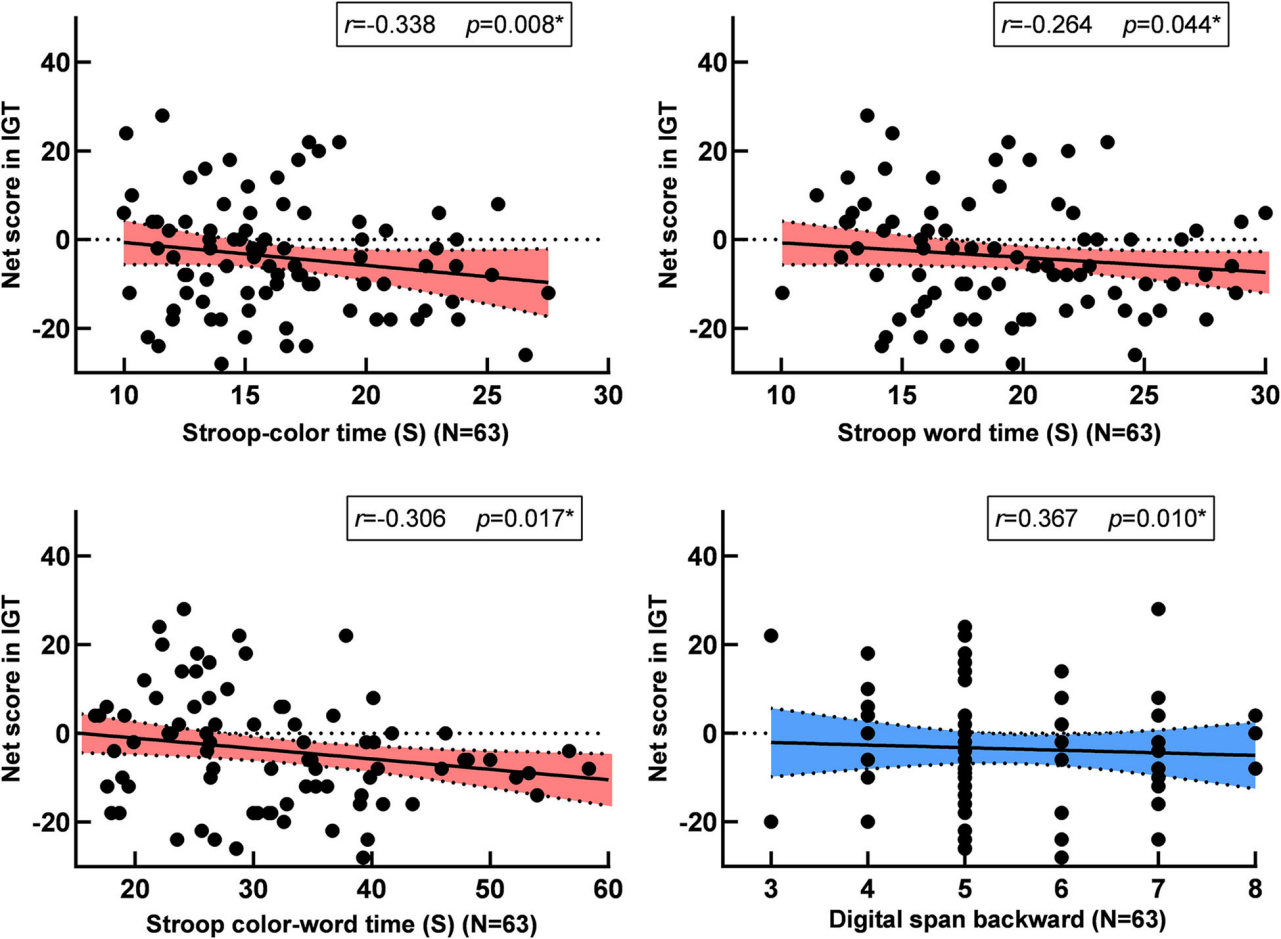
Pearson correlation analyses between the total/net score of IGT, GDT and the performance in the three stroop subtest, digital span backward. The total / net scores of IGT and GDT were respectively negatively correlated with the reaction time in the subtest of the stroop color, stroop word, stroop color-word, they also were positive correlated to the score in the digital span backward. Note: *, *p* < 0.05

Correlational analyses were examined among the performance of DM, the severity of psychiatric symptoms, and the result of neuropsychological measurements in AOS group. Results showed that there was a negative correlation between completion time in the stroop (subtest: Color, Word, Color & Word) and the net/total score of IGT (*p* < 0.05) (Fig. 3). In addition, there was a significantly positive correlation between the score of the Digit Span backward and the net/total score of IGT (*p* < 0.05) (Table 3).

**Table 3:** Pearson’s correlation analyses between the total/net score of DM (IGT and GDT), clinical and neuropsychological data in the AOS (*n* = 63)

Items	IGT Netscore		IGT Total score		GDT Total score		GDT Total score	
	***r*** value	***p*** value	***r*** value	***p*** value	***r*** value	***p*** value	***r*** value	***p*** value
**Stroop Color**	− 0.338^**^	0.008**	−0.343	0.008**	−0.121	0.356	−0.145	0.269
**Stroop Word**	− 0.261	0.044*	−.0360	0.005**	−0.237	0.068	−0.208	0.111
**Stroop Color&Word**	−0.306	0.017*	−0.272	0.037*	−0.060	0.651	−0.140	0.287
**DS - Forward**	0.174	0.233	0.248	0.090	0.113	0.441	0.071	0.626
**DS - Backward**	0.367	0.010*	0.345	0.016*	0.203	0.162	0.131	0.368
**PANSS - Positive**	0.043	0.749	0.244	0.069	0.075	0.580	−0.021	0.879
**PANSS - Negetive**	−0.124	0.378	−0.059	0.676	−0.103	0.462	−0.170	0.222
**PANSS - Total**	−0.111	0.408	−0.015	0.913	−0.076	0.570	−0.197	0.139

Note: *PANSS* Positive and Negative Syndrome Scale. *PANSS-P* PANSS Positive Subscale Score; *PANSS-N* PANSS Negative Subscale Score; *PANSS-T* PANSS Total Score; *DS* Digital Span Test, * p < 0.05, ** p < 0.01

## Discussion

Our findings indicated that patients with AOS showed worse performance than healthy controls in DM, especially under risky condition, as measured by IGT and GDT. In regard to the ambiguous situation, patients with AOS showed significant difference on the 5th block and failed to show learning effect on IGT. Similarly under risk, patients with AOS not only conducted more disadvantageous choices, but also exhibited worse negative feedback rate on GDT. Apart from DM impairment under risk diminished DM abilities under ambiguity were also found to be related to the poor executive function in the present study.

The DM performance in patients with AOS in our present study principally replicated the findings demonstrated by Kester et al. (2006) [38]. Patients with AOS documented a different learning curve compared with their non-psychiatric peers. Although patients with AOS were able to explore an advantageous strategy from the feedback condition on each of the first four blocks, this strategy was incapable of developing a favorable long-term one, and progressed to a high-risk and low-reward strategy on the 5^th^ block. One potential factor may be patients with AOS are implicated with DM dysfunctions under risk, rather than with decisions under ambiguity. Recent evidence has suggested that people had come to learned the rules and contingencies implied in distinct options [34, 49]; and gradually get an awareness of which decks were advantageous or disadvantageous when they performed DM on 3^rd^ or 4^th^ block during the IGT, then making decisions with certainty to the end.

In addition, compared to healthy controls, net scores of the blocks of IGT haven’t shown any difference until progressing to the 5^th^ deck in patients with AOS, while those have showed difference scince  the 3rd or 4th deck in adult patients with schizophrenia [26, 30]. This delay in patients with AOS was probably due to the instability in adolescence [50], characterized by the slow development and the biological changes of the brain regions in the puberty [50–52]. Adolescents are originally sensitive to immediate gains and losses (inhibitory processes), then affecting the further DM strategies on IGT [53–55]. AOS showed comparatively delated DM strategy, coinciding with the adolescent development trajectory [50, 53, 56]. Notably, the prefrontal cortex, with the properties of protracting structural development during adolescence and early adulthood, has been proved as the key brain area of DM, participating in neural processes of DM [50, 52]. Taking into account the alterations of reward processing in the etiology of schizophrenia [52], combining with the late developed impulse control in the adolescence [56], performances of DM in patients with AOS might be consequently damaged. In the future, more researches with a larger sample size and different stages of adolescents with AOS were needed to verify these findings.

In GDT, patients with AOS showed remarkly deficits on risky DM. This result is consistent with the previous studies presented in adolescence patients with neuropsychosis by other scholars [40, 57, 58]. Patients with AOS failed to efficiently integrate consequences and probabilities, and were unable to modify the options from rewards and punishments feedback, possibly lacking a vision on low risk for the higher returns. Our results found patients with AOS not only liked the risky options, but also preferred a higher number in GDT. Similarly, patients with AOS showed worse on processing negative feedback than healthy controls, rather than positive feedback. Patients with AOS were unable to transform a safe/gain option after receiving a big penalty. In other words, AOS preferred to take risks without regard to the consequences of behaviors. Another reason was that AOS may be lack of the ability of impulse control, failing to stop risking actions, and then affecting monitoring and modifying profitable dysfunctions under risk. Previous studies have always stood by the social cognition (especially DM) deficits may be qualified as a hallmark of schizophrenia. However, combining with progressive difference of DM performances occurring only on the 5^th^ block on IGT and high-risk preference in GDT, our results went a step further to indicate that deficits of DM under risk may be a specific socio-cognitive phenotype of AOS.

Results from the current study demonstrated lower score on IGT was related to poor performance executive function in AOS, represented by stroop test and digital span backward. Similarly, blocks and groups interactions were not significant after adding executive function as a covariance on IGT. Executive function plays an inevitable role in DM behaviors, and IGT was called as“hot”executive function in the previous studies [59, 60]. Meanwhile, there is a positive correlation between net/total score on IGT and score on the digital span backward but not the digital span forward in the present study, which may be most likely attributed to the asumption that digital span backward required more cognitive processing, especially depending on the executive resources [61]. These associations concurred with those shown in other previous studies [34, 39, 62]. However, different with previous researches during the DM processes, our present study showed an inconsistent performance that clinical symptoms were unrelated to DM processes. Evidence from previous studies suggested negative symptoms were associated with abnormality of DM processes in schizophrenia through the reinforcement learning [51, 63, 64] and similar results have been found in some fMRI studies. The most probable explanation of these inconsistent results may be that the enrolled patients with AOS in the present study were all in a short course (less than three years), and the possibly limited clinical symptoms may impede the clear presention of the relationship to a certain extent.

In together, a few limitations should be acknowledged. Although the sample size in our present study is larger than any one in the previous published studies of AOS, it is obviously not large enough to be subdivided into different age groups. Second, patients with AOS are given medication during the research. Despite they are all at a stable stage of the illness for more than three month before assessment, we were not able to completely exclude the medicinal impact. The ongoing study should recruit treatment-naive patients to clarify the mechanism of DM deficits under less interference. Third, although the healthy controls were enrolled with psychiatric interview eliminating the history of neuropsychiatric disorders, PANSS was not used to exclude the individuals with mental symptoms. Future studies could be designed including the assessment clinical symptoms.

## Conclusions

Our findings unveiled the abnormal pattern of DM in AOS, mainly reflected on the risky condition, extending knowledge on the performance of DM under ambiguity and risk in AOS. Inefficient DM under risk may contribute to the lagging impulse control and the combined effects of developmental diseases. In addition, our study shows the performance on IGT related to executive function in AOS.

## Supplementary Information

**Additional file 1: Table 1.** Decision-making performance of patients with AOS and healthy controls at different age and gender groups.

## Data Availability

The data are not publicly available due to privacy restrictions but are available from the corresponding author on reasonable request.

## Abbreviations

AOS: Adolescent-onset schizophrenia
DM: Decision Making
IGT: Iowa Gambling Task
GDT: Game of Dice
PANSS: Positive and Negative Syndrome Scale
PANSS-P: PANSS Positive Syndrome Score
PANSS-N: PANSS Negative Syndrome Score
PANSS-T: PANSS total score
DS: The Digital Span Test
