# Supplementary material for: Decision making under ambiguity and risk in adolescent-onset schizophrenia
Source: BMC Psychiatry. 2021 May 4;21:230. doi: 10.1186/s12888-021-03230-1 (PMC8094464; doi:10.1186/s12888-021-03230-1)
Supplement: Supplementary file 1 — Additional file 1: Table 1. Decision-making performance of patients with AOS and healthy controls at different age and gender groups. [file 12888_2021_3230_MOESM1_ESM.doc]

**Supplementary for**

**Decision making under ambiguity and risk in adolescent-onset schizophrenia**

Dandan Li ^a,b,c,d1^, Fengyan Zhang ^e1^, Lu Wang ^a^, Yifan Zhang ^d^, Tingting Yang ^d^, Kai Wang ^a, b,c, d *^, Chunyan Zhu ^b, c, d*^

1. **Decision-making performance of patients with AOS and healthy controls at different age groups**

For the IGT in the 13-15 age group, its main effect among blocks was no significant (*F_4, 164_* = 2.103, *p = 0.093*, *η^2^* = 0.137); The main effect between groups was not significant (*F_1, 41_* = 0.691, *p* = 0.506, *η^2^* = 0.024). There was also no significant blocks×group interaction (*F_4, 164_* = 0.905, *p* = 0.515, *η^2^* = 0.063). For the IGT in the15-18 age group, its main effect among blocks was significant (*F_4, 336_* = 0.353, *p = 0.841*, *η^2^* = 0.012). The main effect between groups was not significant (*F_4, 336_*= 1.496, *p=* 0.228, *η^2^*= 0.024). There was significant blocks × group interaction (*F_1, 83_* =2.24, *p* =0.024, *η^2^* = 0.069).

For the GDT in the 13 - 15 age group, its main effect among numbers was not significant (*F_3, 123_* = 0.713, *p = 0.550, η^2^* = 0.056). The main effect between groups was not significant (*F_1, 41_* = 2.286, *p* = 0.115, *η^2^* = 0.107); There was no significant combination numbers × group interaction (*F_3, 123_* = 2.204, *p* = 0.052, *η^2^* = 0155). For the GDT in the 15-18 age group, its main effect among numbers was not significant (*F_3, 249_* = 2.031, *p = 0.116*, *η^2^* = 0.072). The main effect between groups was not significant (*F_1, 83_* = 0.774, *p=* 0.465, *η^2^* = 0.019). There was significant combination number× group interaction (*F_3, 249_* = 5.008, *p* < 0.001, *η^2^* = 0.162).

**Table 1. Decision-making performance of patients with AOS and healthy controls at different age groups (M**±**SD)**

| **Item** | **Patients with AOS** | **Healthy Controls controls** | ***P* value** | **Patients with AOS** | **Healthy Controls controls** | ***P* value** |
| --- | --- | --- | --- | --- | --- | --- |
| **IGT** | **13-15 years old** | |  | **15-18 years old** | |  |
| **Number of participants** | 25 | 16 |  | 46 | 37 |  |
| Block1 | -2.58±4.88 | -2.07±3.51 | 0.650 | -3.13±4.81 | -4.81±4.4 | 0.040* |
| Block2 | -1.35±3.67 | -0.43±4.09 | 0.363 | -0.45±4.03 | -1.48±4.98 | 0.176 |
| Block3 | 1.03±5.39 | 0.14±6.35 | 0.563 | -0.59±5.18 | -1.07±6.03 | 0.604 |
| Block4 | 1.23±4.72 | 1.04±5.49 | 0.887 | -0.51±6.33 | -0.22±6.13 | 0.824 |
| Block5 | -0.58±7.5 | 1.64±5.61 | 0.206 | -1.31±6.06 | 1.63±5.72 | 0.011* |
| Total net score | -2.26±13.83 | 1.43±15.01 | 0.330 | -5.65±13.75 | -4.59±14.67 | 0.678 |
| Total score | 1496±475.8 | 1557.14±500.27 | 0.632 | 1417.2±5452.74 | 1502.7±8416.71 | 0.281 |
| **GDT** |  |  |  |  |  |  |
| Number 1（N1） | 3.76±3.98 | 1.19±1.56 | 0.006** | 3.85±3.45 | 0.89±1.49 | <0.001*** |
| Number 2（N2） | 3.24±1.9 | 3.81±3.37 | 0.542 | 3.28±2.52 | 3.97±2.89 | 0.249 |
| Number 3（N3） | 4.24±1.9 | 5.63±3.81 | 0.130 | 4.61±2.65 | 6.03±2.43 | 0.014* |
| Number 4（N4） | 6.68±3.85 | 7.38±4.81 | 0.612 | 6.24±3.96 | 7.11±4.03 | 0.327 |
| Total net score | 4.00±8.94 | 8.00±8.55 | 0.161 | 3.65±7.66 | 8.27±6.55 | 0.004** |
| Total score | -24323±991.73 | 168.75±2024.75 | 0.009** | -2171.74±3062.65 | 281.08±1909.16 | <0.001*** |
| Use of negative^a^（%）AAAA  feedback (%) | 0.70±0.24 | 0.82±0.3 | 0.185 | 0.6±0.29 | 0.72±0.27 | 0.045* |
| Use of positive^b^（%）  feedback (%) | 0.56±0.36 | 0.72±0.25 | 0.117 | 0.64±0.31 | 0.69±0.26 | 0.455 |

**p <0.05, **p <0.01, *** p <0.001.*


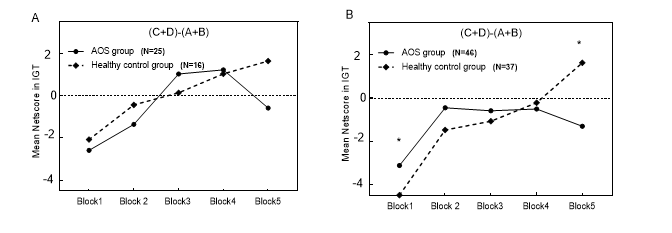


**Figure 1 Iowa Gambling Task (IGT) performance across time between patients with AOS and healthy controls in the age of 13-15 years old (A) and 15-18 years old (B)**

1. No significant net score difference between two groups occurred in the five blocks in IGT.
2. Significant net score difference between two groups occurred on the 1^st^ and 5^th^ blocks in IGT.

Note: ** vs Block 5, *p* < 0.01.


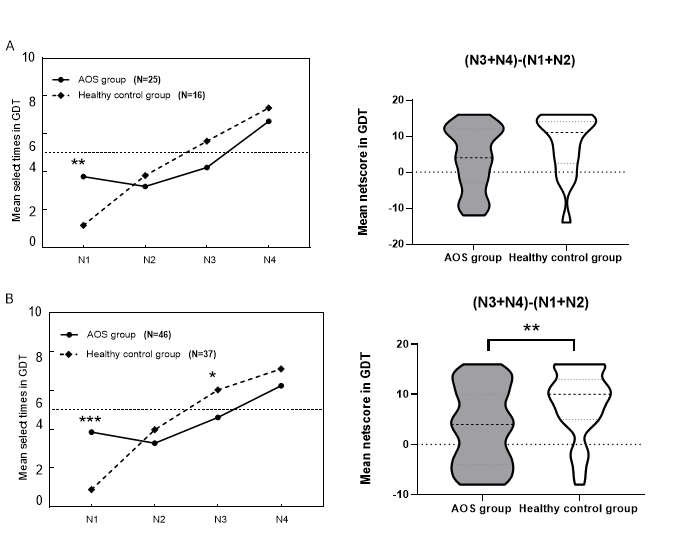


**Figure 2 Performances on the Game of Dice in AOS and healthy controls in the age of 13-15 years old (A) and 15-18 years old (B)**

(A) AOS group were more likely to choose high risk options (N1), Group comparisons revealed that means of the net score in AOS are no significant difference than those in the healthy controls (B) AOS group were more likely to choose high risk options (N1), healthy controls were more likely to choose low risk options(N3), Group comparisons revealed that means of the net score in AOS were lower than those in the healthy controls Note: **p <0.05, **p <0.01, *** p <0.001*

1. **Decision-making performance of patients with AOS and healthy controls at different gender**

The two-factor mixed design analysis was divided into two factors: block/numbers and gender in patients of AOS. Results [shows](C:\\Users\\aa\\Desktop\\新建文件夹\\javascript:;) [that](C:\\Users\\aa\\Desktop\\新建文件夹\\javascript:;) there was no significant block×gender interaction in the IGT (*F*_4, 284_ = 2.015, *p* = 0.098, *η^2^* = 0.076), the same to no significant number × gender interaction in the GDT (*F*_3, 213_ = 0.564 , *p* = 0.652, *η^2^* = 0.008). That is to say, there was no effect of gender on IGT or GDT performance.

1. **IGT and GDT performance between patients with AOS and healthy controls with covariate analysis**

Cognitive function was used as a covariate in group-block/number analysis in the IGT and GDT. We still found significant number × gender interaction in the GDT, but not the IGT. Result as below:

For the IGT, its main effect among blocks was significant (*F _4, 488_* = 7.558, *p < 0.001, η^2^* = 0.201); The main effect between groups was not significant (*F_1, 122_*= 0.615, *p=* 0.542, *η^2^* = 0.010); There was no significant blocks×group interaction (*F _4, 488_* = 1.225, *p* = 0.285, *η^2^* = 0.039).

For the GDT, its main effect among numbers was no significant (*F_3, 366_* = 11.328, *p < 0.001, η^2^*=0.290); The main effect between groups was not significant (*F _1, 122_* = 0.088, *p* = 0.916, *η^2^*= 0.002); There was significant combination numbers×group interaction (*F_3,366_* = 2.286, *p* = 0.015, *η^2^* = 0.091).
